# A proposed model of xeno-keratoplasty using 3D printing and decellularization

**DOI:** 10.3389/fphar.2023.1193606

**Published:** 2023-09-20

**Authors:** Xinyu Wang, Rawdah Taha Elbahrawi, Azhar Mohamud Abdukadir, Zehara Mohammed Ali, Vincent Chan, Peter R. Corridon

**Affiliations:** ^1^ Biomedical Engineering and Healthcare Engineering Innovation Center, Khalifa University, Abu Dhabi, United Arab Emirates; ^2^ Department of Immunology and Physiology, College of Medicine and Health Sciences, Khalifa University, Abu Dhabi, United Arab Emirates; ^3^ Center for Biotechnology, Khalifa University, Abu Dhabi, United Arab Emirates; ^4^ Hleathcare, Engineering and Innovation Center, Khalifa University, Abu Dhabi, United Arab Emirates

**Keywords:** cornea, xeno-keratoplasty, decellularization, recellularization, 3D bioprinting, slaughterhouse waste, bioink

## Abstract

Corneal opacity is a leading cause of vision impairment and suffering worldwide. Transplantation can effectively restore vision and reduce chronic discomfort. However, there is a considerable shortage of viable corneal graft tissues. Tissue engineering may address this issue by advancing xeno-keratoplasty as a viable alternative to conventional keratoplasty. In particular, livestock decellularization strategies offer the potential to generate bioartificial ocular prosthetics in sufficient supply to match existing and projected needs. To this end, we have examined the best practices and characterizations that have supported the current state-of-the-art driving preclinical and clinical applications. Identifying the challenges that delimit activities to supplement the donor corneal pool derived from acellular scaffolds allowed us to hypothesize a model for keratoprosthesis applications derived from livestock combining 3D printing and decellularization.

## 1 Introduction

After cataracts and glaucoma, corneal opacity is the third leading cause of blindness worldwide (Gain et al., 2016). It can be caused by various conditions and infections, resulting in partial or total blindness (Burton, 2009). The treatment for corneal opacity depends on the underlying condition and includes oral and ocular medications, phototherapeutic keratectomy (laser ablation surgery), and keratoplasty (transplantation) (Shibru et al., 2023). Partial or full-thickness keratoplasty techniques include endothelial, anterior lamellar, penetrating keratoplasties, and keratoprosthesis implantation (Armitage et al., 2019). Approximately 180,000 procedures are performed annually worldwide (Gao et al., 2020), yet the supply of corneal grafts is insufficient to meet the current and growing demand for transplantation. For instance, in 2015, there were 10 million patients left untreated worldwide (Wilson et al., 2016), resulting in only 1 in 70 transplants (Gain et al., 2016). Unfortunately, graft recipients often experience several complications resulting from surgical procedures and recovery, which lead to graft failure and rejection. Coupling the supply and demand mismatch with a 35%–70% chance of graft compromise after 2 years of the procedure necessitates the consideration of alternative approaches that simultaneously increase the graft supply and long-term transplantability (Gurnani et al., 2022).

As a result, researchers are creating artificial and bioartificial scaffolds that can be used to develop transplantable constructs alternatives are being developed using artificial and bioartificial scaffolds that can form a basis for transplantable constructs. These efforts date back to reports by Nussbaum, who created the first keratoprosthetic prototype using a quartz crystal and implanted it into the rabbits’ cornea (Holland et al., 2021). Thereafter that, corneal substitutes created from glasses and quartz rimmed with platinum formed the initial generation of keratoprostheses (Kpros). Even though these Kpros could function between 6 and 36 months, they were gradually abandoned due to their heaviness, stiffness, and capacity to induce infections (Holland et al., 2021). These issues drove the need for alternative materials that would be biocompatible and lighter. The discovery of poly (methyl methacrylate) (PMMA), a synthetic artificial material, during the Second World War, fulfilled these important requirements and provided new ways to address corneal degeneration. For instance, Boston Keratoprosthesis was the first group to devise a synthetic keratoprosthesis using a PMMA backplate secured with a titanium locking ring (Holland et al., 2021). However, it does not eliminate the need for donor human corneas. Later on, advancements in the field have led to various synthetic alternatives, such as the Osteo-Odonto-Keratoprosthesis (OOKP), composed of a donor root tooth and alveolar bone to support a PMMA optical cylinder (Sc et al., 2011). This system was combined further with a larger biconvex optic to enhance its design.

Unfortunately, the OOKP procedure is associated with several complications affecting visual outcomes due to its complex surgical technique (Holland et al., 2021). Such issues, along with high costs and prerequisites that limited the patient pool, led to the system being discontinued. In comparison, the rigidity of PMMA ensured that Kpros systems designed from this material required a resilient skirt material to assist the tight attachment with the host eye, leading to further complications (Holland et al., 2021). Therefore, scientists have searched for other materials, like cross-linked poly (2-hydroxyethyl methacrylate) (PHEMA), fibronectin, poly(ethylene glycol) (PEG), and fluorocarbon polytetrafluoroethylene (PTFE) to produce better corneal alternatives or skirts for PMMA-based Kpros (Holland et al., 2021; Vacalebre et al., 2023). Two examples are the Alphacor™, which is composed of PHEMA, and the Korea Seoul-type keratoprosthesis, which contains PMMA and a PEG skirt (Holland et al., 2021). A third example of a synthetic cornea alternative is the most recent addition, the CorNeat KPro. This artificial corneal implantation device is composed of electrospun nanofibers and a biocompatible, nondegradable biomimetic material that imitates the microstructure of the extracellular matrix (ECM). It is a collagen mesh that provides structural and biochemical support to surrounding cells differing from scaffolding and collagen matrices used in tissue repair due to its nondegradable nature. In so doing, this device supports fibroblast migration and colonization, which play a crucial role in wound healing. As expected, *in vivo* studies have shown increased proliferation of fibroblasts and collagen fibrils within several weeks of implantation. Regardless, synthetic corneal replacements are criticized due to multiple complications, including severe levels of inflammation and their non-aesthetic appearance (Fu and Hollick, 2023). Moreover, the currently explored synthetic materials used for generating suitable Kpros are limited.

In comparison, bioartificial corneal scaffolds have been considered effective substitutes for reducing immunogenicity and enhancing compatibility and integration into the recipient (Wilson et al., 2013; Pantic I. V. et al., 2023). As previously stated, the supply of human donor corneas is incapable of meeting existing and projected transplantation needs. Consequently, researchers turned their attention to obtaining replacement corneal tissues from xenogeneic resources. One promising approach to support this process is tissue/organ decellularization, which supports the generation of ECM-rich scaffolds that can be used as templates to create viable corneal substitutes. Decellularization removes the cellular and genetic components of original tissues, thereby decreasing immunogenicity, while maintaining biocompatibility, innate architecture, and various bioactive factors that can drive regeneration and remodeling *in vivo* (Corridon et al., 2017; Corridon, 2021; Wang et al., 2022a; Wang et al., 2022b; Corridon, 2022; Neishabouri et al., 2022; Pantic et al., 2022; Shakeel and Corridon, 2022; Corridon, 2023a; Corridon P., 2023). For instance, Xenia^®^ is a custom-made product derived from decellularized porcine corneas (Islam et al., 2019; Sharifi et al., 2021; Wilson et al., 2022). In clinical settings, these substitutes have reduced the risk for host-immune responses, compared to native (non-decellularized) xenografts, while experiencing appreciable degrees of integration into the implantation site and visual acuity (Wilson et al., 2022). Furthermore, this form of xenotransplantation offers the potential to better balance the supply/demand mismatch for corneal transplantation (Cooper et al., 2007).

Throughout history, numerous attempts have generated Kpros from pigs, sheep, dogs, rabbits, and, more recently, gibbons, cows, and fish (Hara and Cooper, 2011). Remarkably, the cornea is considered an immune-privileged tissue, as it is not immediately vascularized. This characteristic supports its use for xenografting (Cooper et al., 2007). Moreover, recent studies have revealed the potential to repurpose (Dey et al., 2023) and generate a limitless supply of corneal xenografts using slaughterhouse waste (Yoeruek et al., 2012a; Yoeruek et al., 2012b; Pantic I. V. et al., 2023; Khan et al., 2023; Wang et al., 2023). Based on this potential, we assessed current applications and performance in preclinical and clinical practices of various decellularization protocols used to create Kpros and their ability to maintain pertinent structural and physiological capacities. In addition, we propose an alternative corneal xenograft model that may be realized using 3D bioprinting and decellularization technologies. Our evaluations and research-based judgments of synthetic and bioartificial grafts may help establish and guide future research and expedite progress in clinical settings.

## 2 Methods of corneal decellularization

Tissue/organ decellularization procedures effectively remove cellular and nuclear material while maintaining the residual ECM’s biochemical composition and biomechanical integrity to support the development of new tissue. It provides a more effective alternative to ease the constraints of autologous grafting than synthetic vascular tissue engineering procedures (Wilson et al., 2013; Wang et al., 2022b). Eliminating cellular components, and related waste should reduce potential host rejection or immune reaction (Khan et al., 2023). Corneal decellularization aims to yield biocompatible ECM components that possess latent biochemical cues to support *in vitro*/*in vivo* tissue remodeling and long-term transplantability (Wilson et al., 2013; Wang et al., 2022b). Since the corneal stroma has the most organized ECM in the body, tissue architecture, protein, and glycosaminoglycan (GAG) ￼ content maintenance is particularly important. The corneal stroma is a dense, collagen-rich ECM assembled in regularly packed collagen fibrils that are responsible for tissue transparency (Wilson et al., 2013; Chen et al., 2015). The collagen fibrils have a uniform distribution of small (25–35 nm) diameter components assembled in 200–250 nm thick orthogonally stacked layers called lamellae (Crapo et al., 2011; Wilson et al., 2013; Chen et al., 2015). Hence, maintaining this distinctive structure is a crucial part of the corneal decellularization protocols.

**FIGURE 1:**
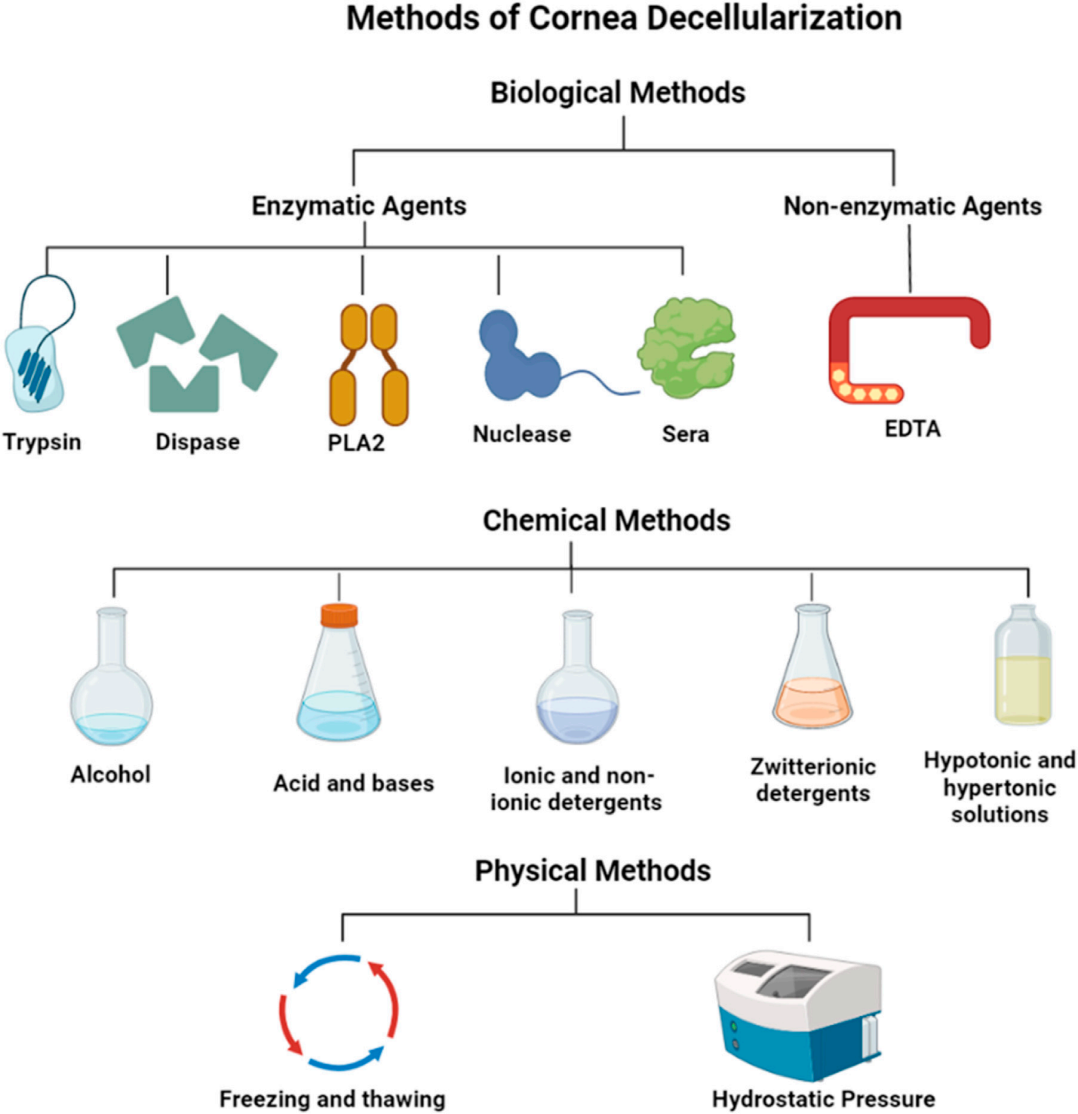
Methods for cornea decellularization.

In general, corneal decellularization involves the breakdown of the cellular membrane followed by processes that separate the cell’s constituents from the ECM. Specifically, cytoplasmic, nuclear components, and cellular debris are removed with detergents and other reagents/processes that disrupt plasma membranes. Afterward, it is necessary to ensure that all residual chemicals have been removed from the tissues. Unremoved decellularizing agents can continue to alter the ECM composition, adversely disrupt the scaffold ultrastructure (Wilson et al., 2013), and generate immunogenic responses during the recellularization process and post-transplantation (Corridon, 2021). Hence, several methods are used in the corneal decellularization process (Table 1; Figure 1) to adequately balance the removal the cellular and nuclear components of the tissue, and the retention of essential structural and bioactive ECM components that support graft develpoment (Crapo et al., 2011; Wilson et al., 2013). Most of these techniques have been examined in bovine, ovine, and porcine corneas (Gusnard and Kirschner, 1977; Amano et al., 2008; Wilson et al., 2013; Pantic I. V. et al., 2023; Khan et al., 2023; Wang et al., 2023). An overview of some commonly used approaches, which can be primarily classified as biological, chemical, and physical, for corneal decellularization and their effects on cellular and extracellular tissue constituents is presented below.

**TABLE 1:** Methods for cornea decellularization and associated mechanisms, advantages, and disadvantages.

Methods/Techniques	Mechanism of action	Advantages	Disadvantages
Biological			
Enzymatic Agents			
Trypsin Zhang et al., 2007; Gilpin and Yang (2017); Isidan et al. (2019)	Hydrolyzes protein and disrupts protein-protein interactions	Breaks cell-matrix interactions	An extended exposure can disrupt the collagen structure
Dispase Gonzalez-Andrades et al. (2011)	Cleaves peptides associated with basement membrane proteins	Can aid the decellularization process by initially removing epithelium and endothelium	May cause damage to the basement membrane
Phospholipases A_2_ (PLA_2_) Wu et al. (2009)	Hydrolyzes phospholipid components of cells	Effective at the removal of DNA and residual cellular components that tend to adhere to ECM proteins	—
		Helps maintain collagen and proteoglycans in the corneal tissue	
Nucleases (RNase and DNase) Cebotari et al. (2010)	Cleaves nucleic acids and aid in their removal	Effective at the removal of DNA and residual cellular components that tend to adhere to the stroma’s ECM proteins	Incomplete removal of the enzymes may impede recellularization and successful transplantation
Sera Wu et al. (2009)	Serum nucleases degrade DNA and RNA.	Effectively removes cells while maintaining tissue transparency	The use of non-human sera carries a risk of cross-species transmission of pathogens
Non-enzymatic Agents			
EDTA Alhamdani et al. (2010)	Dissociates cells by separating metal ions	Can be used for effective when combined with other agents	Ineffective at cell removal when used unaccompanied
Chemical			
Alcohols			
Ethanol Ponce Márquez et al. (2009), Wilson et al. (2013)	Dehydrates and lyses cells	More effective in removing lipids from tissues than lipase	Can cause damage to the ultrastructure of tissue
	Removes lipids from tissues	Antimicrobial, antifungal, and antiviral properties	
Glycerol Lynch and Ahearne (2013), Wang et al. (2022b)	Dehydrates and lyses cells	Can maintain or restore corneal transparency	Can cause damage to the ultrastructure of tissue
	Removes lipids from tissues	Cryoprotectant for long-term cornea storage	
Acids and Alkalis			
Peracetic acid Ponce Márquez et al. (2009), Gilpin and Yang (2017)	Solubilizes cytoplasmic components of cells	Acts to simultaneously sterilize tissue	Ineffective decellularization that can also disrupt the ECM
	Removes nucleic acids via hydrolytic degradation		
Ammonium hydroxide Choi et al. (2010), Dai et al. (2012)	Hydrolytic degradation of biomolecules	Results in complete DC with little effect on collagen architecture	Can eliminate GFs and reduce mechanical properties
Ionic Detergents			
Sodium dodecyl sulfate(SDS) Ponce Márquez et al. (2009), Du and Wu (2011)	Solubilizes cell membranes and dissociates DNA from protein	Complete removal of cells can be achieved	Can be highly detrimental to ECM structure including disorganization of collagen fibrils and loss of GAGs
	Disrupts protein-protein interactions		Loss of tissue transparency
Sodium deoxycholate Blum et al. (2022)	Solubilizes cell membranes and dissociates DNA from protein	Complete removal of cells can be achieved when used with other agents	Less effective at removal of cells
Cebotari et al. (2010), Wang et al. (2022b)	Disrupts protein-protein interactions		
Non-ionic Detergents			
Triton X-100 Cebotari et al. (2010)	Breaks up lipid-lipid and lipid-protein interactions	Mild and non-denaturing	Less effective than ionic detergent treatments
			Can cause damage to the ECM
Zwitterionic Detergents			
CHAPS Alhamdani et al. (2010), Keane et al. (2015)	Has properties of non-ionic and ionic detergents	Better cell removal than non-ionic detergents	Poor cellular removal
		Improved preservation of the ECM ultrastructure than ionic detergents	Very disruptive to stromal architecture
Hypo- and Hypertonic Solutions			
Sodium Chloride (NaCl) Alhamdani et al. (2010), Ekser et al. (2012), Wilson et al. (2013)	Detaches DNA from proteins	Can maintain optically clarity	Does not remove cellular residues
		Ability to maintain the stromal architecture and retain GAG content	Mixed reports on the success of cell removal efficiency
Tris-HCl Alhamdani et al. (2010), Wilson et al. (2013)	Lyses cells by osmotic shock	Reduces decellularization time	Mixed reports on cell removal
Physical			
Freeze-thawing Crapo et al. (2011), Wang et al. (2022b)	Ice crystal formation causes cell lysis	Effectively destroys tissue and organ cells	Expensive
			Needs subsequent treatment to remove cells
			Enhanced pore formation and disruptions to ECM
Hydrostatic Pressure Crapo et al. (2011), Wilson et al. (2013), Gilpin and Yang (2017), Wang et al. (2022b)	Increase in pressure results in cell lysis	Effectively decellularizes while maintaining collagen fibril structure	Expensive
		Kills bacteria and viruses	
Sonication and Mechanical Agitation Xu et al. (2008)	Cell lysis and removal	Does not remove DNA remnants from the corneal tissue	Only effective with enzymatic treatments

### 2.1 Biological techniques for the decellularization of cornea

#### 2.1.1 Enzymatic agents

Enzyme-based treatments for decellularization disrupt the bonds and interactions between nucleic acids, as well as interacting cells in neighboring proteins and other cellular/tissue components (Wang et al., 2022b). These types of treatments are advantageous over other decellularization protocols in that they provide high specificity for removing cellular and detrimental ECM elements (Wilson et al., 2013; Isidan et al., 2019; Mendibil et al., 2020). For example, enzymatic treatments commonly use trypsin, dispase, and phospholipase A2 (PLA2). Trypsin is a serine protease that targets the C-side bonds in arginine and lysine amino acids and is mostly used combined with ethylenediaminetetraacetic acid (EDTA), a chemical agent able to break cell-matrix interactions (Wilson et al., 2013; Mendibil et al., 2020). Extended exposure to trypsin-EDTA treatment can dramatically alter the matrix’s structure by degrading collagen laminin, removing GAGs, and ultimately reducing the tissue’s mechanical strength. (Rieder et al., 2004; Yang et al., 2009). As a result, this combination may not be well suited for corneal decellularization.

In comparison, Dispase II treatment can effectively remove epithelial and endothelial segments (Zhang et al., 2007) before complete decellularization is achieved with a subsequent method. This enzyme degrades peptides linked to basement membrane proteins like collagen IV and fibronectin. Still, if administered over an extended time, it can also harm the basement membrane (Wang et al., 2004). After delipidation of the dermis, a direct comparison of trypsin and dispase treatments showed superior decellularization by dispase accompanied by increased ECM disruption (Prasertsung et al., 2008). Likewise, PLA2 is an esterase that hydrolyses phospholipid components of cells but does not react with collagens or proteoglycans (Wu et al., 2009). Hence, the application of PLA2 in decellularization helps maintain collagen and proteoglycans in the resulting scaffold (Zhang et al., 2007; Wilson et al., 2013). It has also been demonstrated that PLA2 and sodium deoxycholate ￼ were influential in producing the acellular porcine corneal scaffolds (Wu et al., 2009; Azevedo et al., 2018). Other studies have also suggested that combining PLA2 with a bicarbonate salt can support effective cellular removal and maintenance of collagen fibers (Wu et al., 2009; Li et al., 2011).

Furthermore, concerning combinative treatments, nucleases are mainly applied with other detergents to expedite the removal of DNAs and RNAs from scaffolds (Grauss et al., 2005; Heath, 2019; Neishabouri et al., 2022). RNases and DNases are frequently used to cleave nucleic acids and aid in removing nucleotides after cell lysis in tissues (Crapo et al., 2011). For instance, porcine corneas treated with DNase and RNase resulted in efficient decellularization, but the tissue became opaque due to severe distortion of the collagen structure (Oh et al., 2009). However, other studies have identified, in many cases, as a general consequence of decellularization, and optical clearing agents like glycerol can be used to reverse opacity (Bochert et al., 2005; Polisetti et al., 2021; Hedhly et al., 2022; Wang et al., 2023) while providing antimicrobial benefits (Lin et al., 2012; Gupta and Upadhyay, 2017; Chaurasia et al., 2020). Compared with exonucleases, endonucleases such as benzonase (Gonzalez-Andrades et al., 2011) may be more effective because they cleave nucleotides mid-sequence and thereby more effectively remove DNA fragments (Wilson et al., 2013). Likewise, another category of enzymes, such as sera-derived enzymes, including fetal bovine serum, contains nucleases that can degrade DNA and RNA (Gui et al., 2010). They support the removal of nucleic acids from tissues but fail to remove immunogenic elements (Crapo et al., 2011). The xenogeneic serum may also introduce immunogenic elements into the ECM, which can cause adverse responses following recellularization or transplantation (Crapo et al., 2011; Shao et al., 2012). Moreover, the human serum has also been used as a standalone decellularizing agent to produce porcine decellularized cornea (Armitage et al., 2019), after first mechanically removing the epithelium (Shao et al., 2012).

#### 2.1.2 Non-enzymatic agents

In contrast, non-enzymatic treatments include the use of chelating agents and serine protease inhibitors. Chelating agents such as ethylenediamine tetra-acetic acid (EDTA) aid cell dissociation by separating metal ions (Crapo et al., 2011; Wilson et al., 2013; Isidan et al., 2019). However, these mechanisms can disrupt protein-protein interactions (Crapo et al., 2011). Most notably, chelating agents alone are incapable of adequate cellular removal. Thus, they are often used in combination with enzymes and detergents (Crapo et al., 2011). EDTA has also been used with sodium dodecyl sulfate (SDS), a potent ionic detergent, to decellularize corneal tissues effectively (Bayyoud et al., 2012).

In comparison, serine protease inhibitors, like aprotinin, phenylmethylsulfonyl fluoride, and leupeptin, can prevent some detrimental effects to the ECM caused by intracellular proteases released after the cellular lysing process (Wilson et al., 2013). Specifically, protease inhibitors often accompany harsh detergents and decellularizing agents. One common agent used for corneal decellularization is aprotinin (Wilson et al., 2013), an inhibitor of trypsin and related proteolytic enzymes. In studies conducted with these agents, authors have reported minimal damage to the ECM despite the use of harsh decellularizing agents (Du and Wu, 2011; Yoeruek et al., 2012a). A summary of enzymatic and non-enzymatic decellularization techniques is presented in Table 1 below.

### 2.2 Chemical techniques for the decellularization of cornea

#### 2.2.1 Acid and base treatments

As one of the most common chemical substances for decellularizing, acids and bases cause or catalyze the hydrolytic degradation of biomolecules. Acids were found to dissociate nuclear DNA from ECM by disrupting nucleic acids and solubilizing cytoplasmic components. Additionally, solutions with extreme pH levels were indicated to be highly effective in the decellularization process (Neishabouri et al., 2022). For instance, it has been shown that increasing the pH of the zwitterionic agent, cholamidopropyl dimethyl ammonio-1- propane sulfonate (CHAPS), during decellularization increases the effectiveness of cell and protein removal (Neishabouri et al., 2022). Moreover, increasing the pH of a compound could also eliminate growth factors and disrupt the mechanical structure of the scaffold. Overall, acid and alkali treatments effectively solubilize cytoplasmic components and eliminate nucleic acids (Gilbert et al., 2006), by catalyzing hydrolytic degradation of biomolecules (Crapo et al., 2011). However, such solutions may also degrade essential bioactive molecules such as GAGs from collagenous tissues. To illustrate this point, using potent acids to facilitate decellularization has resulted in the retention of sulfated GAGs, but the damage and removal of collagen from scaffolds, thereby reducing the structural integrity of ECM (Crapo et al., 2011; Neishabouri et al., 2022).

Apart from CHAPS, peracetic acid is a highly corrosive and commonly used disinfectant and oxidizing agent in sterilization (Gilpin and Yang, 2017). It doubles as a decellularization agent by removing residual nucleic acids with minimal effect on the ECM composition and structure (Crapo et al., 2011). Unfortunately, limited success has been reported with corneal tissue when used alongside ethanol; however, Kao et al. considered further optimization was necessary to improve its utility (Wilson et al., 2013). In addition, peracetic acid has been reported to retain GAG content and preserve the structure and function of essential growth factors (Gilbert et al., 2006).

In addition to acids, ammonium hydroxide, as an alkaline-based treatment, has been used in conjunction with the detergent Triton X-100 to decellularize human corneas (Azevedo et al., 2018). This treatment combination effectively decellularized the tissue with little apparent effect on the collagen architecture and basement membrane proteins (Vacalebre et al., 2023). Nevertheless, ammonium hydroxide is also known to degrade growth factors and adversely alter the mechanical properties of the ECM.

#### 2.2.2 Alcohols

Another class of decellularizing agents is alcohols. The mechanism by which these compounds decellularize tissues is based on their ability to dehydrate tissues, and ultimately lyse cells (Gusnard and Kirschner, 1977; Crapo et al., 2011). Alcohols such as ethanol and isopropanol are commonly used to degrade lipids, integral components of the plasma membrane (Wilson et al., 2013). They are more effective in removing lipids from tissues than lipases (Crapo et al., 2011). Previous reports have shown that ethanol treatments have resulted in complete corneal decellularization while maintaining the overall tissue structure (Ponce Márquez et al., 2009). Interestingly, corneal stromal cells cultured on ethanol-treated decellularized corneas (DCs) were reported to proliferate slower and produce more new ECM components compared to those cultured on DCs produced using detergents (Ponce Márquez et al., 2009). However, care should be taken while using alcohols as a decellularizing agent, as they can act as a tissue fixative (Jamur and Oliver, 2010) that can crosslink or precipitate proteins (Jamur and Oliver, 2010), damage the ECM ultrastructure (Crapo et al., 2011), and disrupt collagen fiber alignment, leading to corneal opacity (Lynch and Ahearne, 2013).

Apart from ethanol and isopropanol, glycerin dehydration has been used since the 1960s for prolonged storage of donor corneas for later transplantation (Wilson et al., 2013). More recently, *in vivo* confocal microscopy techniques have shown that antigen-presenting cells and stromal cells were, in fact, absent in glycerin-cryopreserved allografts (GCA) used in transplantation procedures into human recipients (Chen et al., 2010). These DCs showed promising results when transplanted into patients but were also plagued by a high risk of rejection due to infection and inflammation (Li et al., 2011). Afterward, in a clinical study, no rejection was seen with the GCA, while only 10% of eyes that received fresh corneal allografts (FCA) reported episodes of stromal rejection (Li et al., 2011). Another clinical study reported similar findings without rejection cases in the GCA group and one case of stromal rejection in the FCA group (Chen et al., 2010). These findings suggest that glycerol cryopreservation may be a promising technique for producing DCs for use in corneal grafting procedures, with apparent clinical efficacy. Glycerol has also been used as a post-treatment to restore corneal transparency (Alhamdani et al., 2010) and as a preservation technique for corneal decellularization.

#### 2.2.3 Surfactants

By far, the most employed methods of decellularization are based on surfactants. Surfactants are chemical agents used to solubilize cell membranes and dissociate their inner structure. These agents also dissociate DNA from proteins, and they are, therefore, effective in removing cell materials from tissues (Crapo et al., 2011). Surfactants are classified into three genres: ionic, non-ionic, and zwitterionic. For example, polyethylene glycol (PEG), an amphiphilic, non-ionic copolymer that damages cell membranes and has been shown to satisfactorily decellularize pig and human corneas (Yoeruek et al., 2012a). In comparison SDS, an anionic surfactant agent, rapidly ablates cells and cell membranes (Wang et al., 2022b). SDS is widely used for corneal decellularization, yet the protocols still need to be standardized (Isidan et al., 2021). According to the reported studies, Zhou et al. employed 0.1% (wt./vol.) SDS for cornea decellularization that lasted 7 h at 37°C (Zhou et al., 2011; Isidan et al., 2019), whereas Gonzalez-Andrades et al. recommended to immerse corneas in 0.1% (vol/vol) SDS for 48 h at room temperature accompanied with 300 rpm successive shaking (González-Andrades et al., 2015; Isidan et al., 2019). These procedures resulted in varying degrees of decellularization.

Similarly, zwitterionic detergents, like 3-[(3-cholamidopropyl dimethylammonio]-1-propane sulfonate)] (CHAPS), have been applied in both immersion and perfusion decellularization procedures (Gilpin and Yang, 2017). Zwitterionic detergents possess properties of both ionic and non-ionic detergents and have been shown to better support cell removal from native tissues than non-ionic detergents and preserve the ECM ultrastructure than ionic detergents (Hudson et al., 2004; Gupta et al., 2018; Heath, 2019). However, CHAPS has been criticized as cytoplasmic proteins and cell fragments were retained in stromal tissue scaffolds, indicating ineffective decellularization (Gilbert et al., 2006; Du et al., 2011; Wilson et al., 2013). When used for corneal decellularization, poor cellular removal, as well as damage to ECM proteins have been reported (Du et al., 2011). Except for CHAPS, some surfactant-treated samples will unfortunately succumb to detrimental structural disruptions, namely, the unwanted removal of valuable ECM proteins, as evidenced by proteomic studies (Gui et al., 2010; Crapo et al., 2011; Wilson et al., 2013). For example, using Triton X-100 to facilitate decellularization is not appropriate for tissues where GAG and lipid retention are essential (Gilpin and Yang, 2017). Triton X-100 can often denature cell membranes without denaturing proteins, however at higher concentrations this non-anion detergent can break protein -protein interactions and degrade tissue architectures, while providing lower decellularization efficiencies and degree of transparencies, than ionic agents like SDS (Yoeruek et al., 2012a; Wilson et al., 2013). As a result, to decellularize the cornea it has been used in combination with other agents, including hydroxylamine (Gonzalez-Andrades et al., 2011), and Dispase II, for the removal of the corneal epithelium (Wilson et al., 2013) and nucleases to aid in the complete removal of cellular debris. In the latter cases, these confounding factors have made assessing detergent efficiency and its effects on the ECM challenging. However, in all cases, adequate levels of decellularization were reported, and the tissue’s ultrastructure and mechanical properties were well preserved following the decellularization process (Wilson et al., 2013; Gilpin and Yang, 2017).

#### 2.2.4 Hypotonic and hypertonic solutions

Excluding the abovementioned chemical treatments, hypertonic and hypotonic solutions can also disrupt cell stability owing to osmotic pressure generated between internal and external tissue membranes, resulting in cellular lysis (Mendibil et al., 2020; Wang et al., 2022b). Yet, this lysing process is not known to remove the cellular residues that it releases into the ECM, which reduces this process’s effectiveness as a decellularization technique. Other encouraging studies have demonstrated that appreciable decellularization efficacies can be obtained with other chemical reagents since they do not disturb ECM composition (Neishabouri et al., 2022). For instance, the use of hypertonic sodium chloride (NaCl) solution has been investigated for its ability to generate DCs (Gonzalez-Andrades et al., 2011). Results from such studies have shown that the hypertonic solution caused minimal damage to stromal architecture and retained many extracellular growth factors and proteoglycans (Shafiq et al., 2012). As a result, the DCs produced from this method retain optical clarity after decellularization. Moreover, using tris-HCl buffers as a pretreatment at 4°C, to initially lyse cells before further treatment with previously mentioned reagents has the advantage of reducing the incubation time needed in the harsher decellularizing techniques (Wilson et al., 2013).

### 2.3 Physical techniques for the decellularization of cornea

#### 2.3.1 Freeze-thaw cycles

Physical technologies are also considered an essential component in decellularization protocols. The application of the freeze-thaw cycle has been shown to effectively degrade cells in tissues via the formation of intracellular crystals (Crapo et al., 2011). Freeze-thaw cycles are generated by fluctuating between freezing (−87°c) and normal biological (37°C) temperatures (Wang et al., 2022b; Neishabouri et al., 2022). Such fluctuations help frozen water crystals occupy significant volumes inside cells and thus cause their membranes to rupture. This process aids a uniform degree of decellularization (Mendibil et al., 2020; Rabbani et al., 2021). However, varied crystalline geometries may damage the scaffold and alter the mechanical stability of the ECM (Keane et al., 2015; Wang et al., 2022b). Experiments by Xaio et al. utilized snap freezing followed by lyophilization to induce pore formation in DCs. The ice crystals formed during pre-freezing were sublimated under vacuum conditions, leaving a network of interconnected pores that enabled cellular infiltration (Wilson et al., 2013). Pulver et al., 2014 (Pulver et al., 2014), Xing et al., 2015 (Xing et al., 2015), and Rahman et al., 2018 (Azevedo et al., 2018) have also shown that multiple freeze-thaw cycles can be used for decellularization (Neishabouri et al., 2022). Freezing and incubating the tissue in nitrogen gas has been used to induce apoptosis, as freezing alone can be insufficient (Wilson et al., 2013). Furthermore, nitrogen freezing of tissues is a relatively mild, yet costly treatment, compared to enzymatic or detergent treatments (Wilson et al., 2013).

#### 2.3.2 High hydrostatic pressure

High hydrostatic pressure (HHP) processes disrupt cellular membranes within the corneal tissue by generating isostatic pressures. Pulses of water are sprayed on tissues immersed in saline to generate acellular scaffolds (Rabbani et al., 2021; Wang et al., 2022b). High hydrostatic pressure is non-cytotoxic and successfully removes cells while destroying bacteria and viruses (Lynch and Ahearne, 2013). Studies have demonstrated that this decellularization approach can be more effective than detergents or enzymes while providing a comprehensive platform for recellularization, even though the ice crystal formation may alter ECM ultrastructure (Crapo et al., 2011; Rabbani et al., 2021). However, to prevent non-ideal structural alterations in the ECM, the amount of force applied needs to be accurately managed (Wang et al., 2022b). High hydrostatic pressure has been reported to successfully decellularize porcine corneas while maintaining the collagen fibril matrix and GAG content (Wilson et al., 2013). Nevertheless, this procedure is costly, as it requires specialized equipment to induce pressures of up to 1 GPa that are needed to generate viable acellular corneal tissues (Lynch and Ahearne, 2013).

#### 2.3.3 Sonication and mechanical agitation

Unlike HHP and freeze-thaw cycles, which can be applied independently, sonication and mechanical agitation are combined with chemical and enzymatic treatments for decellularization (Gilbert et al., 2006). For example, a porcine retinal specimen was completely decellularized using a hydrostatic pressure of 980 MPa for 10 min in a study by Hashimoto et al. (Azuma et al., 2007). The aorta of a pig sample was likewise the subject of a similar study. In both instances, a chemical agent was required to destroy and remove DNA remnants from the tissue because the physical approach alone could not do so (Rabbani et al., 2021). As a result, sonication, and mechanical agitation work better when used with chemical and enzymatic decellularization agents.

Overall, the corneal decellularization process entails removing cellular and nuclear components while preserving the biochemical composition and structural integrity of the ECM for tissue remodeling and long-term transplantability. Various biological, chemical, and physical methods are used for this process. Biological techniques involve enzymatic agents such as trypsin, dispase, nucleases like DNase and RNase, and PLA2. In addition, non-enzymatic agents like chelating agents and serine protease inhibitors are used for corneal decellularization. Chemical procedures employ acids and alkalis to break down biomolecules, such as peracetic acid and ammonium hydroxide. These methods include ionic, non-ionic detergents, zwitterionic detergents, and hypotonic and hypertonic solutions.

Moreover, physical methods such as freeze-thawing, hydrostatic pressure, sonication, and mechanical agitation are also utilized in the process. Each method has advantages and disadvantages regarding cell removal efficiency, effectiveness in degrading biomolecules, and preservation of ECM structure.

## 3 Characterization of decellularized cornea prostheses

Various techniques can create corneal scaffolds through decellularization. Meanwhile, each has pros and cons, targeting distinct components of tissues/cells to form scaffolds with different properties, which necessitates their characterization. To create corneal scaffolds with favorable biological and mechanical performances, characterizations of these substitutes before *in vivo* studies are extremely crucial. Such characterizations involve the assessment of the cellular removal efficacy, maintenance of structure and function of scaffolds, biocompatibility, biodegradability, the potential for remodeling, cytotoxicity, pathogenicity, and immunogenicity within the post-transplantation environment (Hussein et al., 2016).

Accordingly, incomplete removal of cellular materials can trigger unfavorable host responses as well as associated cytocompatibility issues (Brown et al., 2009; Nagata et al., 2010). Hence, the protocols for decellularizing the cornea should be monitored to discern whether complete decellularization, including removal of all cellular debris, has occurred and ECM integrity has been maintained after processing (Wilson et al., 2013). As a further step, characterizations can be done to determine the retention of the native ECM components and their resulting mechanical and physiological characteristics. This approach is carried out because ECM is extremely pivotal for sustaining the normal structure and function of the corneas, and no other tissues of the human body rely more on ECM and its components than corneas (Espana and Birk, 2020). Except for the biocompatibility and biodegradability, the prerequisite for *in vivo* grafting, the functionalization characterizing of decellularized cornea grafts such as the ability of cell repopulation and anti-infections, mechanical properties like pressure resistance, elastic stiffness, and optical features including a viable anterior refractive power for the eye and levels of high transparency are quite critical for clinical applications (Sridhar, 2018). As a result, standardized metrics and approaches are required. Additionally, an ability to routinely gauge decellularization efficacy efficiently and consistently would support future manufacturing purposes (Wilson et al., 2013). Computational and artificial intelligence techniques may provide pivotal support in this area (Corridon et al., 2022; Davidovic et al., 2022; Pantic et al., 2022; Shakeel and Corridon, 2022; Pantic et al., 2023b; Pantic et al., 2023c; Pantic et al., 2023d; Valjarevic et al., 2023). For this study, we will now discuss the characterization of decellularized Kpros in detail by focusing on ways to cellular removal, scaffold biocompatibility, preservation of innate biological attributes, and scaffold recellularization.

### 3.1 Removal of cellular materials

It has been established that residual DNA in scaffolds is a primary contributor to adverse host reactions (Brown et al., 2009; Nagata et al., 2010), The associated scaffold characterizations are focused mainly on the removal of DNA. Effective decellularization criteria currently require ECM to contain <50 ng of dsDNA per mg dry weight, DNA fragments shorter than 200bp, and no visible nuclear components (Crapo et al., 2011). Techniques that facilitate such characterizations rely on conventional histological and biochemical methods. Brightfield microscopy and hematoxylin-based DNA staining protocols are widely used for evaluating nuclear debris, and it is typically employed in conjunction with eosin to evaluate the fundamental ECM architecture of DCs (Sc et al., 2011; Fu and Hollick, 2023; Vacalebre et al., 2023). Fluorescent nuclear stains such as DAPI (Du and Wu, 2011; Gonzalez-Andrades et al., 2011; Shafiq et al., 2012), Hoechst (Amano et al., 2008; Wu et al., 2009; Corridon, 2022; Corridon, 2023c), and propidium iodide (Shafiq et al., 2012) are also frequently utilized.

By fluorescently labeling the terminal end of nucleic acids, assays like the TUNEL assay, which detects levels of DNA fragmentation, can be used to measure cell death throughout the decellularization process (Amano et al., 2008). DNA staining and imaging are the standards for assessing decellularization, used throughout the body of literature, relatively insensitive and offer a means to gauge decellularization efficiency quantitatively. Additional quantitative information can be extracted from various spectrophotometric assays (Gui et al., 2010). Residual DNA fragment sizes can also be assessed by gel electrophoresis. However, the disadvantage of performing these assays and staining protocols to assess residual DNA is that the destruction of the sample is required (Wilson et al., 2013).

As complete preservation of ECM ultrastructure is ideal, decellularization protocols aim to minimize its disruption. Unfortunately, there is no reliable reagent or standardized protocol that can achieve complete decellularization (Crapo et al., 2011; Wilson et al., 2013; Isidan et al., 2019), as the term “complete decellularization” is currently not well defined (Crapo et al., 2011). Hence, the term “sufficient decellularization” or “effective decellularization” are suggested (Isidan et al., 2019). These terms refer to the minimal criteria that relate to an adequate threshold of native component removal since removing 100% of cellular material while maintaining a viable ECM is impossible.

### 3.2 Biocompatibility evaluation of corneal scaffolds

Following the confirmation of the effective eradication of cellular materials, evaluating the biocompatibility of the acellular tissues and organs is critical to limit the potential to induce adverse *in vitro* and *in vivo* consequences. These evaluation studies should be performed based on the rules and guidelines of the International Standards Organization (ISO) (Hussein et al., 2016). Regarding the biocompatibility evaluation, several techniques are presented. To examine the cytotoxicity of corneal replacements, assays are applied to monitor compounds and characterize the latent harmful effects of scaffolds before clinical use. One of the major parameters considered is an *in vitro* cytotoxicity evaluation, which measures cell culture viability qualitatively and quantitatively (Keong and Halim, 2009). Such studies are essential since the main concerns are related to the risks using decellularizing and sterilizing agents. For instance, using SDS for decellularization could damage the ECM and inhibit future recellularization (Singer and Tjeerdema, 1993). Likewise, sterilizing processes are required to eliminate harmful antigens in scaffolds, so that implantation regimens can generate minimal or untraceable immunogenic responses (Crapo et al., 2011; Kajbafzadeh et al., 2013). However, sterilizing procedures can denature the scaffold structure, and the ineffective removal of harmful foreign agents can induce toxicity (Hussein et al., 2016). Therefore, it would be indispensable to test the cytocompatibility after sterilization.

The other concern is the cytotoxicity. Cytotoxicity results in significant functional and structural damage due to a cascade of molecular events that interfere with macromolecular synthesis (Murray et al., 2007). Studies used to evaluate cytotoxicity expose segments of scaffolds to distinct cell culture lines. Identifying the characteristic signs of toxicity shown by cells makes it possible to determine whether cells can thrive in the scaffold environment. (Granchi et al., 1998). Cytotoxicity is preferred to be tested *in vitro* since cells cultured *in vitro* are generally more sensitive to toxic substances than *in vivo* tissues. *In vitro* cytotoxicity evaluations are often preferred (Cenni et al., 1999), and the following two types of assessments are typically employed: indirect contact essay and direct contact essay.

Current methods assume that cytotoxicity is studied on a cellular level rather than the molecular level, meaning the components of decellularized scaffolds will elicit their overall effects at the cellular level (Hussein et al., 2016). This realization indicates that additional techniques, such as mRNA microarray, should be considered to provide an enhanced perspective on this issue (Hussein et al., 2016). Specifically, mRNA microarray techniques can provide an unbiased insight into all transcripts, thereby supporting the detection of adverse molecular components that induce injury (Rao et al., 2018).

In addition to the cytotoxicity, pathogenic problems that may arise from decellularization have raised concerns since decellularized tissues or organs primarily emanate from cross-species. These species typically carry the paramount virus, bacteria, and other potentially dangerous pathogens. For instance, the U.S. Food and Drug Administration (FDA) banned the use of nonhuman primates for xenotransplantation due to the enhanced risk of disease transmission (Butler, 1999). Fortunately, advances in gene editing may be able to circumvent these challenges (Arabi et al., 2023), along with mandatory testing before xenotransplantation (Hayat et al., 2019), as the risks cannot be avoided completely (Michaels et al., 2001; Fishman et al., 2012). Furthermore, more research is required to identify pathogens and facilitate their effective removal (Fishman et al., 2012).

Besides pathogenicity, immunogenicity is also a complicated problem that requires addressing for decellularization and grafting. It is mandatory to avoid hyper-acute and acute rejections to ensure the biocompatibility of xenografts *in vivo* transitions (Naso et al., 2012). This issue relates to the ultimate removal of cellular components, antigens, nucleic materials, immunogenicity tests, and the current ability to completely void a tissue of potentially immune components. Regarding the immunogenicity assessment, *in vitro* inflammatory response mainly uses macrophage and lymphocyte cell lines with indirect or direct contact assays to observe the immune performance of macrophages (Groell et al., 2018). *In vivo*, inflammatory studies have been investigated by diverse quantitative assays. Methods to quantify infiltrated leukocytes at the transplantation site and phenotyping cellular infiltrates and cytokine profiles are commonly applied (Orlando et al., 2012; Mirmalek-Sani et al., 2013; Park et al., 2013). New techniques, including mRNA microarray and proteomics, will also enable investigations of the interactions between cells and decellularized tissues, referring to a response to surface-adsorbed proteins and the proteins expressed from reseeded cell signaling (Hussein et al., 2016). Such studies can improve our understanding of cell-scaffold interactions, ultimately providing ways to enhance recellularization efforts.

### 3.3 Preservation of innate biological attributes post-decellularization

After the adequate removal of cellular materials and biocompatibility evaluations of decellularized scaffolds are appropriately conducted, the next step should be to perform characterizations that provide insight into how the decellularization protocol can preserve the structural and functional characteristics of the native tissues. An overview of various characterization techniques used to evaluate decellularized corneal scaffolds is outlined in Table 2.

**TABLE 2:** A summary of characterization techniques used to evaluate decellularized corneal scaffolds.

Mechanical techniques	Application	Advantages	Disadvantages
Compression testing Stammen et al. (2001)	The tissue is placed under two plates and compressed	It can measure the mechanical behavior of the tissue and ductile fracture limits of the tissue	It flattens the tissue and damages the structural architecture
	The test is used to determine the mechanical behavior of the tissue under the crushing load	It also gives you a detailed assessment of the tissue’s load-bearing capacity and elasticity properties	Due to the corneal curvature shape, the test may not distribute the pressure equally
Holographic interferometry Calkins et al. (1981)	Is a tool that uses a laser to trace the changes in the tissue and perform interferometric measurements	It is a precise method that can detect residual stresses and cracks on the tissue without mechanical contact	There is no fixed distance so the location of the structure cannot be obtained
Bulge and inflation testing Stammen et al. (2001)	Is a tool that is used to biomechanical test corneal tissue by inflating the tissue and measuring the displacement	It is a reliable tool that demonstrates the intrinsic properties of the cornea layers and resembles Intraocular pressure	The inflation capacity is difficult to control and it could affect viscoelasticity
Corvis STL Tonometer/Pachymeter Wang et al. (2022c)	Corvis ST is a device that uses a high-speed Scheimpflug camera to record the cornea movement	Corvis ST device evaluates the central cornea thickness, corneal stiffness, and intraocular pressure	Very expensive
Ultrasound	It is a device that uses sound waves to get a very detailed image of structures	It is a non-invasive technique that shows detailed surface imaging of the cornea	It depends on the user’s skill
Ocular Response Analyzer Kaushik and Pandav (2012)	Is a non-invasive device that uses rapid air pulse to make an indentation in the cornea	It measures the cornea biomechanical properties such as corneal hysteresis, intraocular pressure, and corneal resistance factor	Very expensive
Indentation testing Wilson et al. (2013)	Is a test that measures the indentation left behind in the cornea after it was compressed	Determine the hardness of the cornea with minimal destruction	Doesn’t assess tensile strength

#### 3.3.1 ECM architecture preservation

Although the preservation of the native tissue architecture and ECM composition during decellularization is crucial for all tissues, it is of particular interest to corneas. This interest stems from the cornea’s highly organized ECM, which is responsible for its primary function, light transmission (Musselmann et al., 2006). Various histological assays can be used to compare the architecture of DCs with the native cornea. Studies have shown that stains such as eosin and van Gieson’s can adequately reveal alterations in collagen structure (Du et al., 2011; Du and Wu, 2011). However, basic histological approaches do not always provide sufficient specificity, and under these circumstances, immunohistochemistry is a helpful tool since it is possible to identify ECM proteins specific to the cornea (Wilson et al., 2013). Corneal-specific proteins typically include collagens I, II, III, IV, and V, keratin, fibronectin, and laminin, in the basement membrane (Choi et al., 2010; Du and Wu, 2011). Ideally, an intact Bowman’s layer and Descemet’s membrane is a significant goal post-decellularization. This goal is often evaluated by identifying specific proteins rather than using a broad eosin stain, which will provide additional information. Stains such as Alcian blue have been used to identify other scaffold components to assess whether the GAG content within the corneal stroma has been retained (Gonzalez-Andrades et al., 2011; Wilson et al., 2013).

#### 3.3.2 Transparency

The corneal stroma is mainly composed of dense connective tissue (Meek and Knupp, 2015). The cornea protects the inner content of the eye, and the refractive capability of the lens is based on the precise shape of this region (Teng et al., 2006). This region can be subdivided into various layers, including the epithelium, Bowman’s layer, stroma, Descemet’s membrane, and endothelium. Optical transparency and clarity are two characteristics that determine vision quality. Assessing the transparent nature of corneal tissue is very challenging, and techniques are limited. Previously, the transparency was assessed subjectively by placing the cornea on top of a grid and evaluating how the lines were seen. Luckily, throughout the years, the quantitative measurement of cornea tissues has improved using various methods, and each technique has advantages and disadvantages. Below is a discussion of various conventional and emerging microscopic techniques that can measure and monitor corneal transparency.

Conventional light microscopy is a simple technique that uses visible light to detect small objects. Even though this approach offers appreciable resolution assessments (Morishige et al., 2007), a major drawback is the need to introduce dyes, which can distort the specimens, to enhance visualization. In contrast, confocal microscopy offers higher-resolution optical imaging evaluations that can provide more insight into finer structures like corneal nerves *in vivo* and assess cell organelles with better precision (Jalbert et al., 2003; Corridon et al., 2013; Hall et al., 2013; Collett et al., 2017; Corridon et al., 2017; Kolb et al., 2018; Corridon et al., 2021; Cai et al., 2022; Shaya et al., 2022; Corridon, 2023d). In a previous study, confocal microscopy was used to assess the structural architecture of a human cornea. The Bowman’s membrane, stroma, and endothelium were all visualized at a 1–2 μm resolution. Such a high resolution could be obtained by limiting the field of view to a single spot and thus eliminating out-of-focus structure (Jalbert et al., 2003). A drawback of this tool is that it can only precisely analyze a small part of the specimen and provide a limited field of view, so it will take a long time to analyze multiple regions. Confocal microscopy can reconstruct a full view by rapidly scanning the cornea. However, its utility as a screening tool is limited because it cannot fully assess the quality of the ECM or evaluate collagen organization after decellularization.

In comparison, transmission electron microscopy uses beams of electrons to visualize the ultrastructural details of the cornea and generate high-resolution magnified images (Gusnard and Kirschner, 1977). This form of microscopy was used to assess rabbit cornea stroma opacity. Researchers were able to achieve remarkable images of the regeneration of the epithelial basement membrane (Torricelli et al., 2013). However, a drawback of the technique is that it uses powerful fixatives, which could again distort the specimen and affect decellularization.

Alternatively, anterior segment, Fourier-domain optical coherence tomography (Goswami et al., 2019) is a non-invasive tool that uses light waves to take cross-sectional images of the eye. It Is essential for diagnosing a broad spectrum of diseases that affect the eye, such as glaucoma, macular degeneration, and diabetic eye disease (Drexler et al., 2001), as observed with other forms of comparable spectroscopic applications for highly accessible regions of the body (Corridon et al., 2006). It can provide microstructure information such as stromal thickness, stromal morphology with an axial resolution, and the Bowman layer, providing another tool to assess decellularization. In comparison, ultrasound biomicroscopy uses a probe that emits high-frequency sound waves (50–100 MHz) that reflect through the tissue and generate an image (He et al., 2012). It Is useful for diagnosing a broad spectrum of diseases that affect the eye and giving an accurate, detailed image of the cornea. This form of microscopy provides advantages over the previously mentioned techniques as it does not require staining and can delineate the microarchitecture and measure variations in the corneal layer. Nevertheless, this approach is limited by its need for direct contact with the eye and low (4 mm–5 mm) tissue depth penetration (Alexander et al., 2021).

#### 3.3.3 Biomechanical properties

Recall that decellularization is a process that removes the cellular and nuclear material from the tissue while trying to maintain the remaining innate composition and the biological and mechanical properties of the tissue (Lynch and Ahearne, 2013). Some decellularization protocols can damage the cornea by changing the 3D architecture of the tissue, altering the biomechanical properties, and reducing the transparency of the cornea (Lynch and Ahearne, 2013). Historically, quantitative measurements of cornea tissue biomechanical properties have improved. We can now measure the cornea intraocular pressure, load-bearing capacity, elasticity properties, central cornea thickness, and stiffness. However, further means are needed to assess the effect decellularization protocols could have on the viscoelastic properties of the cornea (Lynch and Ahearne, 2013). Below is a tabular summary of standard mechanical techniques and their advantages and disadvantages in assessing the biomechanical properties of the cornea.

### 3.4 Evaluation of recellularization performance

Beyond the initial structural and functional assessments of acellular corneal grafts, the cell reseeding performance of these scaffolds should be emphasized and required before *in vivo* implantation. Recellularization is a dynamic process that aims to repopulate the decellularized scaffold. For this process, it is essential to determine the benefits and drawbacks of the different cell lines (Joseph et al., 2016). Previous research has shown the feasibility of using immortalized cell lines such as induced pluripotent stem cells (iPSCs) (Joseph et al., 2016). iPSCs can be differentiated into keratocyte cells that could be used to repopulate the scaffold (Joseph et al., 2016). Stem cells enable the development of unlimited cell sources that can be used for various therapeutic purposes. They also offer the opportunity for autogenic cell transplantation, but the significant problem is that the iPSCs have the potential to proliferate uncontrollably and form teratomas (Corridon et al., 2017).

Other restrictions to corneal recellularization are stroma thickness and the dense packing of collagen fibrils. Thus, it can be challenging for the cells to repopulate the decellularized scaffold if they cannot reach their target. One technique injects the cells into the stroma directly, but a significant drawback to this approach is that it can damage the 3D architecture (Villalona et al., 2010). Another cell seeding technique coats the scaffolds with biological glues to enhance cellular adhesion and eventually penetrate and migrate into deeper layers. This technique can increase cell seeding efficiency (Villalona et al., 2010). Research has shown that different techniques are needed, depending on the site of cornea damage; for example, patients with limbus damage develop limbal stem cell deficiency (Fernández-Pérez and Ahearne, 2020). This damage leads to the patient developing conjunctivalization, which leads to corneal opacity and loss of vision. To treat this disease, some studies have used limbal-derived stem cells (Fernández-Pérez and Ahearne, 2020). These stem cells are isolated and transplanted onto the cornea to proliferate and replenish the limbus population. A successful procedure is assessed based on the patient’s lack of immune reaction, transparency recovery, and corneal restoration.

There is a need to conceive specific methods to drive and evaluate the recellularization of the decellularized corneal scaffold segments (Nouri Barkestani et al., 2021). Namely, recellularization approaches using keratocytes alone may need concurrent treatments to support regeneration []. Furthermore, work by Martin et al. has presented the differentiation of human embryonic stem cells (hESCs) into corneal epithelial-like cells on the decellularized cornea matrix has been demonstrated for the first time, which implied the potential employment of hESCs on the corneal substitutes to support repopulation. Decellularized corneal technologies have a huge potential to alleviate the cornea shortage. At the same time, the current research on recellularization techniques, protocols, and reseeding cell types needs to be more proficient to support clinical translation. Therefore, it is imperative to enhance recellularizing strategies.

## 4 Preclinical and clinical applications

In the previous paragraphs, we focused on discussion on the relevant biological and biomechanical, functional, and structural characterizations of kerato-alternatives relevant and the associated approaches to generating decellularized replacements. Notwithstanding the need for further research, Kpros have been evaluated in preclinical and clinical settings. In the subsequent sections, current and emerging commercial options are fully elucidated and summarized in Table 3.

**TABLE 3:** A summary of clinical and preclinical studies.

Pre-clinical study		
Study	Overview	Conclusions
CorNeat KPro: Ocular Implantation Study in Rabbits Litvin et al. (2021)	Study performed to evaluate surgical feasibility and long-term integration of the synthetic cornea CorNeat KPro in rabbits	The synthetic KPro integrated with retention of 87.5% at 6-month follow-up period
		Optical element remained clear with no incidence or retro prosthetic membrane formation Litvin et al. (2021)
		Histopathology was comparable to tissue and cellular in all eyes with presence of fibroblasts and associated collagen fibrils within the skirt component
Therapeutic efficacy of mesenchymal stem cells for the treatment of congenital and acquired corneal opacity Call et al. (2019)	Study performed to evaluate the therapeutic efficacy of human umbilical cord mesenchymal stem cells [UMSC] in the treatment of corneal opacity that is associated with collagen V loss in mice	Recovered some corneal transparency in injured corneas of wild-type, Col5a1f/f and Col5a1∆st/∆st mice
	UMSCs transplanted via fibrin gel following the keratectomy, and analyzed at 7 days and 14 days post-transplant via *in vivo* confocal microscopy [HRT]	UMSCs reduced the corneal opacity in the control Col5a1f/f and Col5a1Δst/Δst mice following injury. The Col5a1Δst/Δst mice did not show improvement in corneal haze until 14 days compared to 7 days in the Col5a1f/f mice
Anti‐Inflammatory and Anti‐Fibrotic Effects of Human Amniotic Membrane Mesenchymal Stem Cells and Their Potential in Corneal Repair Navas et al. (2018)	The purpose of this study was to examine whether stem cells obtained from human amniotic membranes [hAM-MSC] can reduce inflammation and fibrosis in corneal chemical burns	*In vitro* assays showed that hAM-MSC CM can diminish human keratocyte differentiation and reduce the release of NETs by human-derived neutrophils. Thus, supporting the theory that hAM-MSC possess anti-fibrotic and anti-inflammatory properties
Transplantation with Cultured Stem Cells Derived from the Human Amniotic Membrane for Corneal Alkali Burns: An Experimental Study Zeng et al. (2014)	The purpose of this study was to examine through an experimental model in rabbits the use of extracted mesenchymal stem cells (MSCs) from human amniotic membranes and the utilization of hAM-dMSCs transplantation, AM grafting, and their combined use in the treatment of alkali burns	Compared with the control group, the treated groups demonstrated faster reconstruction of the corneal epithelium. Further, the control group exhibited corneal opacity scores that were significantly higher than those observed in the other three groups (*p* < 0.01)
A comparison of the effectiveness between amniotic membrane homogenate and transplanted amniotic membrane in healing corneal damage in a rabbit model Guo et al. (2011)	The study aimed to determine whether amniotic membrane homogenate is as effective in healing corneal damage as amniotic membrane transplantation in a rabbit model	Number of layers of neoformative epithelium on the seventh day after surgery was found to be significantly higher in the amnion homogenate group (median = 5.5; IQR = 5.6) and amnion transplant group (median = 5; IQR = 5.6) when compared to the control group (median = 3; IQR = 3, 3.75) (*p* < 0.001)
Effect of amniotic membrane transplantation on the healing of bacterial keratitis Barequet et al. (2008)	The study aimed to use rats as an animal model to examine the efficacy of amniotic membrane (AM) transplantation as adjunctive treatment in corneal healing after bacterial keratitis	It was found that the best clinical results were in the group treated with cefazolin and AM transplantation. They had the least corneal haze and neovascularization (*p* = 0.007 and *p* = 0.014, respectively) and minimal bacterial counts (28 colony-forming units [CFU]/mL compared with 160 CFU/mL and 240 CFU/mL, respectively)
		Histopathology showed that the central corneal vessels from rats treated with cefazolin and AM were smaller and less congested than those from the other two groups
On the influence of neutrophils in corneas with necrotizing HSV-1 keratitis following amniotic membrane transplantation Barequet et al. (2008)	The study aimed to examine whether necrotizing herpetic stromal keratitis (HSK) in mice will improve after amniotic membrane transplantation (AMT)	Reduced severity of corneal keratitis after 2 days of AMT (1.2 ± 0.8 vs. no transplanted 3.1 ± 1.1)
Transplantation of amniotic membrane in murine herpes stromal keratitis modulates matrix metalloproteinases in the cornea Heiligenhaus et al. (2005)	The study aimed to study matrix metalloproteinases (MMP) and tissue inhibitors of metalloproteinases (TIMP) in the corneas from mice with ulcerative herpes stromal keratitis (HSK) treated with amniotic membrane transplantation (AMT)	Severity score of stromal keratitis Improved from 4.0 ± 0.0 on Day 4 of after HSV to 3.1 ± 1.1 2 days after in the control group vs. to 1.2 ± 0.8 2 days after AMT

Clinical Study		
Study	Overview	Conclusions
First in Human (FIH) Study to Assess Safety and Efficacy of the CorNeat KPro for the Treatment of Corneal Blindness Litvin et al. (2021)	Study enrolled 40 participants and the CorNeat KPro is to be implanted unilaterally with follow up procedures being performed at day 1, week 1, and months 1,2,3,6, 9, and 12 following implantations. Clinical assessment of the keratoprosthetics will be done through examination of the eye using slit-lamp biomicroscope, intraocular pressure measurement and ocular imaging Litvin et al. (2021). Further, participants’ visual acuity will be assessed and recorded through the 13 months follow up period	There are no posted results of the ongoing study. One patient who underwent corneal transplants had visible improvement of the corneal repair
Safety and Indicative Effectiveness of Porcine Corneal Lenticular Implants in Patients with Advanced Keratoconus and Post Lasik Ectasia: A Retrospective Clinical Study Wilson et al. (2022)	A retrospective clinical study aimed at o evaluating the safety and feasibility of implanting decellularized porcine corneal lenticules in a femtosecond laser-assisted pocket for patients with advanced keratoconus and post-Lasik ectasia	Visual acuity improved in all patients except for one case at 6 and 12 months (*p* = 0.002 and 0.007). At 1-year follow-up, the mean central corneal thickness increased from 389.43 ± 45.41 to 429.33 ± 63.20 µm, the maximum keratometry decreased from 64.8 ± 5.11 to 62.82 ± 6.16 D, the mean corneal resistance factor (CRT) increased from 5.67 to 8.42, and the total higher-order aberrations decreased from 1.80 to 1.16
	Implantation occurred in 7 eyes with six due to advanced keratoconus and clear cornea and one with advanced post-Lasik ectasia followed for 12 months	
Combined Platelet Rich Plasma and Amniotic membrane in the treatment of Perforated Corneal Ulcer Abdelghany et al. (2022)	This is a case series of 10 patients that were diagnosed with corneal perforation and underwent emergency surgical procedure for repair through implantation of synthetic amniotic membrane with platelet-rich plasma clot under it with application of platelet-rich plasma eye drops - follow-up for 4 weeks	Within the first 7 days all cases demonstrated formation of adequate intraocular pressure digitally, within, and complete sealing of the corneal perforation within the 4 weeks follow up period
		Mild symptoms like foreign body sensation and lacrimation were reported only in the 1st postoperative week
		Three of the patients underwent penetrating keratoplasty after 6 months with satisfactory visual outcomes
Recurrent pterygium treatment using mitomycin C, double amniotic membrane transplantation, and a large conjunctival flap Monden et al. (2018)	The retrospective case series aimed to evaluate the clinical outcomes of surgery for recurrent pterygia using mitomycin C (MMC), double amniotic membrane transplantation (AMT), and a large conjunctival flap	Mean follow-up period was 3.6 years
	Study included 31 eyes in 31 patients with recurrent pterygia. All patients underwent pterygium excision, application of MMC, double AMT, and placement of a large conjunctival flap	Mean preoperative and postoperative best-corrected visual acuities (logMAR conversion) were 0.23 and 0.13, respectively
	Outcome was based on measured visual acuity, astigmatism, and recurrence	Evidence of a significant difference between the mean preoperative (−3.85 D) and postoperative (−2.22 D) astigmatism
	They defined recurrence as the presence of fibrovascular proliferative tissue crossing the limbus	The recurrence rate was 3.2% (1/31 cases)
Surgical result of pterygium extended removal followed by fibrin glue-assisted amniotic membrane transplantation Liu et al. (2017)	This prospective interventional cohort study enrolled 57 patients (58 eyes) who are undergoing pterygium extended removal followed by fibrin glue-assisted amniotic membrane transplantation and report the recurrence rate, complications, and cosmetic results of conjunctival wound edge and caruncle	The cohort included 48 eyes with nasal pterygium, 5 eyes with temporal pterygium, and 5 eyes with double pterygium. 81.0% (n = 47), 0% (n = 0), 12% (n = 7), and 7% (n = 4) of eyes with Grades 1–4 recurrence, respectively
	All patients received postoperative follow-up for at least 12 months	Cosmetic results of conjunctival wound edge and caruncle in cases with nasal pterygium resulted in 59.3% (n = 32), 14.8% (n = 8), 9.3% (n = 5), 16.6% (n = 9), and 0% (n = 0) of eyes with Grades 1–5 morphology respectively
		Overall, 5.1% (n = 3), 3.4% (n = 2), 3.4% (n = 2), 3.4% (n = 2), 1.7% (n = 1), 6.9% (n = 4), and 1.7% (n = 1) of patients suffered from postoperative pyogenic granuloma, transient diplopia, permanent motility restriction, steroid glaucoma, fat prolapse, sub amniotic membrane hemorrhage, and early detachment of amniotic membrane, respectively Litvin et al. (2021)
A study protocol for a multicenter randomized clinical trial evaluating the safety and feasibility of a bioengineered human allogeneic nanostructured anterior cornea in patients with advanced corneal trophic ulcers refractory to conventional treatment González-Andrades et al. (2017)	This is a phase I-II, randomized, controlled, open-label clinical trial of ten Spanish hospitals to evaluate the safety, feasibility, clinical efficacy evidence of a bioengineered human allogeneic nanostructured lamellar anterior cornea in adults with severe trophic corneal ulcers refractory to conventional treatment or with sequelae of previous ulcers	Results pending
Suture less cryopreserved amniotic membrane graft and wound healing after photorefractive keratectomy Vlasov et al. (2016)	This prospective nonrandomized control trial aimed to evaluate the effect of sutureless cryopreserved amniotic membrane (Prokera) on corneal wound healing after photorefractive keratectomy (PRK)	The amniotic membrane graft sped corneal reepithelialization 1 day after PRK but was not better than the bandage contact lens in hastening complete reepithelialization of the cornea. Visual outcomes, corneal clarity, and optical quality of the cornea were comparable between the amniotic membrane graft eyes and bandage contact lens eyes
	Forty patients were enrolled	
Amniotic Membrane Graft for Syntomathic Bullous Keratopathy (AMBUK) Pires et al. (1999)	The study performed amniotic membrane transplantation at 5 centers on 50 consecutive eyes (50 patients) with symptomatic bullous keratopathy and poor visual potential	The follow-up period of 33.8 weeks (3–96 weeks) after amniotic membrane transplantation revealed that 43 (90%) of 48 eyes with intolerable pain preoperatively became pain free postoperatively
		From the 5 eyes that had residual pain, 3 received repeated amniotic membrane transplantation, 1 required a conjunctival flap for pain relief, and 1 had reduced pain
		Epithelial defects in 45 (90%) of 50 eyes created and covered by amniotic membrane healed rapidly within 3 weeks

Keratoplasties have existed since 2000 BC; however, one of the first fully described keratoprosthetic dates to 178 (Holland et al., 2021). Different keratoprosthetic devices from rigid polymers or soft structures can be better integrated into the transplantation site. The Boston Keratoprosthesis is an example of a rugged synthetic keratoprosthesis with a poly (methyl methacrylate) (PMMA) base that has a backplate of PMMA with a titanium locking ring to secure the backplate that was FDA approved in 1992 (Avadhanam et al., 2015). Donor corneal tissue is placed in between the anterior PMMA layer and the backplate; thus, it does not eliminate the need for human donor corneas. The retention rates were around 90%, with post-operative visual acuity of 20/100 or better at 6 months and 1 year in 67% and 75% of patients, respectively. However, corneal melting and graft detachment can occur due to poor biointegration between the PMMA and the corneal stroma (Holland et al., 2021).

Similarly, the OOKP was introduced in 1963 and comprised a donor root tooth and alveolar bone to support a PMMA optical cylinder (Sc et al., 2011). It was later improved in 1998 by adding a larger biconvex optic and performing cryo-extraction of the lens to become known as the modified osteo-odonto-keratoprosthesis (MOOKP) (Gulati et al., 2016). A significant drawback of MOOKP is that it requires a complex surgical technique and patient counseling with complications involving the mucosa, retina, and lamina that affect visual outcomes. Retention failure of the device is commonly due to limited resorption, as there must be a balance between resorption and reformation to preserve the lamina. In contrast, the AlphaCor™ keratoprosthetic is an example of a soft keratoprosthetic composed of a cross-linked PHEMA that forms a hydrogel by polymerization that forms the optical and skirt components (Chirila, 2001). The skirt has a higher water content than the optical component, allowing it to have larger pores for biointegration (Gurnani et al., 2022). It was reported that retention rates for the AlphaCor™ have been relatively high, as seen in a phase I trial where 93% of 14 devices were retained for up to 2.5 years (Crawford et al., 2002). However, due to the higher water content, there can be inadequate suturing performance and poor mechanical strength that allows for stomal melts and extrusion.

Although all the listed solutions have succeeded, future work should deeply involve targeting solutions that reduce the use of human donor tissue through optimizing decellularized cornea scaffolds. One of the strategies is to replicate the corneal microenvironment, which is targeted by applying biopolymers to mimic ECM components. For example, a bioengineered corneal implant was documented as a solution for patients with a high risk of graft failure. The bioengineered implant was made from recombinant human collagen type III (RHCIII). In a phase 1 clinical trial, a biosynthetic cornea of RHCIII crosslinked with non-toxic zero-length human crosslinkers. It was found that there was the regeneration of corneal epithelium, partial replacement of corneal stoma, facilitation of nerve regeneration, and good biointegration with the biomimetic cornea (Fagerholm et al., 2010). However, the biomimetic corneal replacement was only suitable for low-risk patients as it led to neovascularization in rabbit models with severe pathological consequences (Hackett et al., 2011). Thus, efforts were made to reduce the risk of implant-related neovascularization by including a synthetic phospholipid methacryloylocyoxyethyl phosphorylcholine (MPC). The RHCIII-MPC implants prevented vascularization in a high-risk alkali burn corneal injury model. Further, the device was implanted in three patients with ulcerations, decreased corneal integrity, and near blindness, and it was shown that the implants improved vision in two out of the three patients, but in all three patients, there was no evidence of neovascularization at the 1-year follow-up.

More decellularized corneal implants have been explored due to the possibility of optimizing or improving actual corneas’ complex structure and function (Brunette et al., 2017; Fernández-Pérez and Ahearne, 2020). There is a wide variety of decellularization methods, with no superior method identified. However, all methods aim to achieve the same goal. The goal of decellularization of donor corneal tissue is to maximize the elimination of cellular and immunogenic components to decrease the chances of rejections while limiting impairment to the histoarchitecture and mechanical properties of the conserved ECM (Wilson et al., 2022). In addition, decellularization of the tissue should not hinder the ability to repopulate the scaffold with host cells. One example of a method for decellularization includes biological treatments.

Biological treatments are enzyme-based procedures that disrupt the bonds and interactions between nucleic acids and interacting cells by disrupting neighboring proteins and other cellular components. By using enzymatic agents in the decellularization process, it is possible to eliminate cell residuals and other undesirable components of the ECM due to the high specificity that enzymatic agents provide compared to other decellularization protocols. However, using enzymatic agents can result in residual enzymes in the decellularized tissues, impairing the recellularization while stimulating an immune response, including apoptosis and inflammation, which result in early rejection (Crapo et al., 2011; Wang et al., 2022b).

Non-enzymatic agents, including chelating agents and serine protease inhibitors, can also be used, whereby chelating agents such as ethylenediaminetetraacetic acid (EDTA) aid cell dissociation by separating metal ions (Wilson et al., 2013; Wang et al., 2022b). However, these approaches can lead to the disruption of protein-protein interactions (Rieder et al., 2004; Xing et al., 2015). Chelating agents alone are insufficient for superficial cell removal. They are often combined with enzymes and detergents, as unaccompanied, they are insufficient for superficial cell removal (Crapo et al., 2011). Biological treatments are just one example of possible strategies to decellularize effectively. However, as indicated, it poses its disadvantages in successful recellularization.

The emergence of biomaterials and bioinks can be used to mimic the corneal microenvironment. In addition, corneal bioengineering through 3D bioprinting has become an appealing method for developing a corneal equivalent. Rebuilding a stromal equivalent has been attempted by a research team that bioprinted corneal stromal keratocytes (CSK) using collagen-based bioinks (Duarte Campos et al., 2019). Another research group constructed a 3D anatomically analogous corneal structure using an existing 3D digital human corneal model and a composite bioink comprised of collagen and alginate containing encapsulated corneal keratocytes. While the keratocytes remained viable for 7 days post-printing, the metabolic activity and protein expression were low (Isaacson et al., 2018). These are just a few of the attempts at 3D bioprinting of a corneal equivalent, yet many of the attempts did not have any evidence of testing it in implantation.

As mentioned, Xenia^®^ corneal implants aim to reinforce biomechanically compromised corneas with evidence of an increasing number of patients undergoing the treatment with over 12 months of follow-up (El-Massry et al., 2021). Contemporary means of creating more viable kerato-alternatives are intended to combine decellularization with fabrication techniques and simulate the ECM structure to a large extent. There are two appropriate instances for illustrating the solutions. The development of Xenia^®^ lenticules is comprised of a four-stage process. This process begins with decellularizing the corneal tissue, removing the cells, antibodies, and antigens of the donor tissue to decrease the host’s immune system response post-implantation. After the donor tissue is decellularized, it is washed, compressed, and crosslinked, allowing for the reduction of the thickness and increasing the stiffness of the lenticular region, further assisting in the stabilization and reshaping of the keratoconus cornea (Elsheikh et al., 2008). Examination of the effectiveness of developing the Xenia^®^ lenticule on mechanical strength to further examine the load-deformation response to pulsatile, physiologically representative pressure variations showed that the mechanical properties were not homogenous across the tissue, and there was also substantial variability in the calculated corneal stiffnesses (Elsheikh et al., 2008). Although the current use and implementation of Xenia^®^ corneal implants showed promising results, the sample size was small, and the follow-up was not prolonged (El-Massry et al., 2021).

Previous attempts outline the difficulty in developing artificial Kpros due to the formation of postoperative retroprosthetic membrane, corneal melt, limited implant retention, postoperative glaucoma, and overall poor postoperative visual acuity. On the other hand, the CorNeat KPro is a synthetic alternative to bioartificial corneal implants. The undercut on the device’s posterior side lets it easily snap into a trephined cornea and quickly seal the eye. The lens is surrounded by a nondegradable porous integrating skirt, which is fabricated through electrospinning and will be implanted subconjunctivally. The porous property of the skirt allows the conjunctiva to adhere to the sclera employing bio-stitching. The implanted skirt material stimulates cellular proliferation and colonization, contributing to full tissue integration. The CorNeat KPro is combined with a biocompatible, nondegradable biomimetic material that imitates the microstructure of the human ECM. The collagen mesh provides structural and biochemical support to surrounding cells, differing from scaffolding and collagen matrices used in tissue repair due to its nondegradable nature.

**FIGURE 2:**
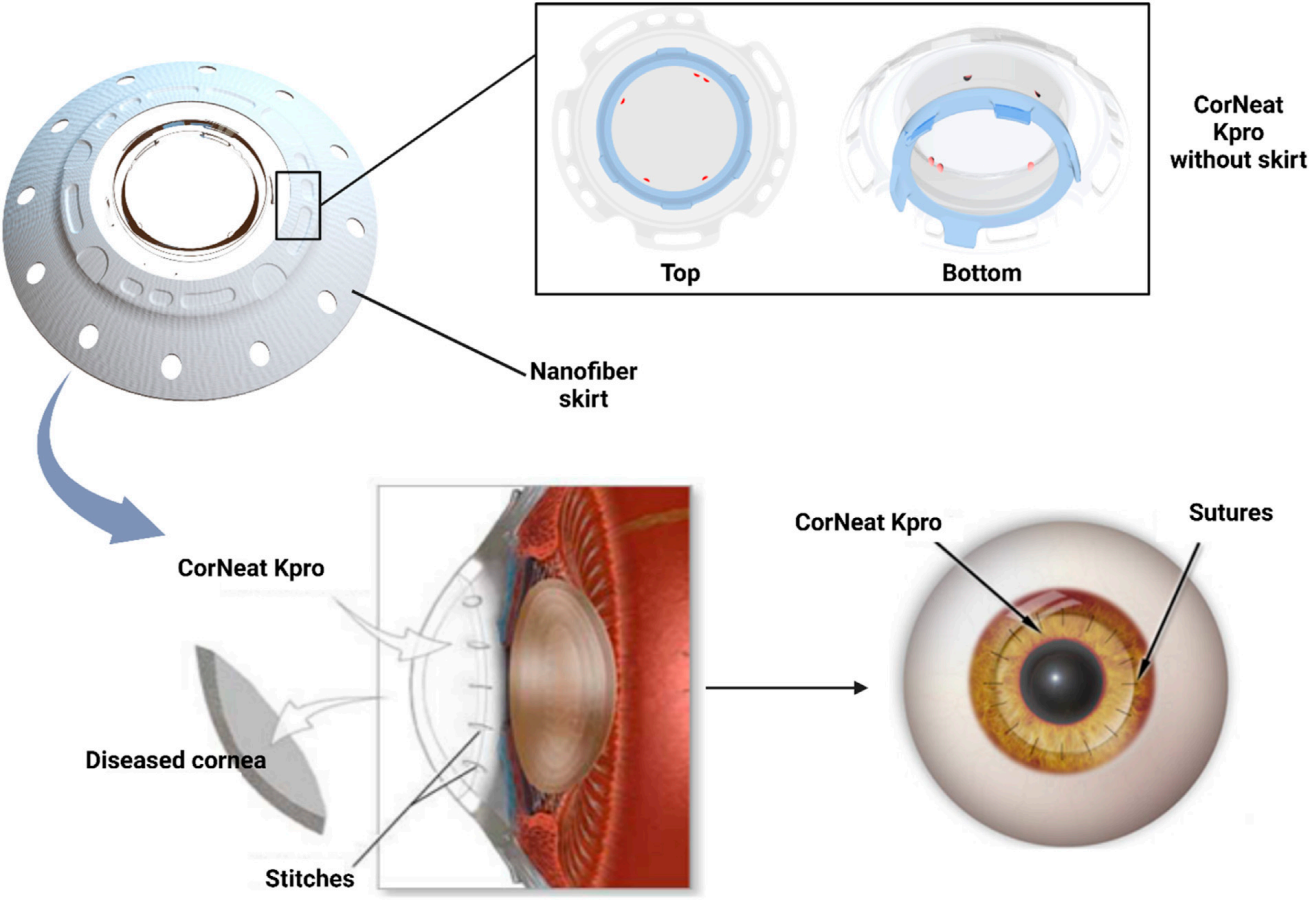
Composition of CorNeat KPro and the implantation process.

Furthermore, this manufactured matrix was designed to provide human fibroblasts with a familiar environment that could support migration and colonization, which are crucial for wound healing and remodeling (Neishabouri et al., 2022). The *in vivo* studies have shown increased proliferation of fibroblasts and collagen fibrils within several weeks of implantation, indicating progressive tissue integration. A schematic of the novel design and implantation procedure is outlined in Figure 2.

## 5 Conclusion and a potential model of keratoprosthesis

Decellularized Kpros have been developed rapidly in recent years and gained more positive feedback and support owing to their minimal immunogenicity, abundant xeno-resources, superior biocompatibility, and mimicked ECM structure. Distinct decellularization techniques were explored and applied to create corneal analogs with different biomechanical performances. Characterizing decellularized kerato-alternatives requires several studies and advanced technologies. Maintaining ECM-similar structure, biocompatibility, and eradicating immunogenic components are the fundamental requirements to produce cornea scaffolds. Scaffolds supporting adequate cellular repopulation and, eventually, long-term transplantation call for promising alternatives to meet existing challenges. Nevertheless, limited decellularization protocol standardization, biocompatibility assessments, proficiency in evaluating biomechanical features, and degree of long-term *in vivo* somatic tissue growth are significant challenges that must be addressed. Therefore, we hypothesized a new model of future Kpros using 3D printing and decellularization techniques.

The native cornea has a transparent and multilayered structure. The primary function of the cornea is to refract and transmit light toward the lens and, ultimately, the retina (Sridhar, 2018). Each layer of the cornea is distinct in composition, structure, function, and resident cells (Dua et al., 2013). The outer layer-epithelium consists of stratified epithelial cells, while the Bowman’s layer and a basement membrane are acellular collagen separating the epithelium from the stroma. Bowman’s layer consists of tiny collagen fibrils with a diameter of 18–22 nm (Birk et al., 1986; Gordon et al., 1994). Bowman’s collagen types are composed of IV and VII, which play essential roles in mechanical performance. The stroma, constituting 90% of the total corneal thickness, contains highly structured, evenly spaced, and anisotropically aligned collagen fibrils (Gipson et al., 1987). The collagen fibrils in the stroma are quite different from that in Bowman’s layer. They are majorly collagen type I. The dimension of the stroma of human corneas is 0.5–250 μm in width and up to 2.5 μm in thickness (Komai and Ushiki, 1991). These collagen fibrils are surrounded by GAGs, glycoproteins, and proteoglycans (Quantock et al., 2015). The stroma contributes to the transparency, avascularity, and mechanical properties needed to maintain the structure (Holland et al., 2021). Moreover, the stroma also includes keratocytes that help maintain homeostasis after an injury, stem cells located in the limbal stromal region which can differentiate into various cells when simulated, and dendritic cells, the most efficient antigen-presenting cells initiating the adaptive immune response (Kawakita et al., 2005; Davies et al., 2013).

The Descemet membrane, filled with hexagonal collagen VIII, IV, and XII networks, is connected with the endothelium, whereas the endothelium inner layer has a single layer of endothelial cells that can regulate the hydration of the whole cornea (Fitch et al., 1990). The Descemet’s membrane supports communication between the corneal endothelium and posterior stroma keratocytes. Ocular swelling and vision impairment are caused by endothelial damage and unregulated angiogenesis (Penn et al., 2008). Since the ECM is not only the pivotal component of corneas but also supports the structure intactness and functional regulation (Hassell and Birk, 2010). Impairment to the ECM will result in reduced vision or even complete visual loss (Maurice, 1957). The cornea’s transparency is determined by the regular packing of the homogenous small collagen fibrils (Meek and Leonard, 1993). Different species have different diameters, amounts, and structures of collagen fibrils (Birk and Trelstad, 1984). Collagen type I, GAGs, glycoproteins, and proteoglycans are major components of the cornea ECM. However, corneal stroma structure and ECM structure do not generally rely on the collagen type (Isaacson et al., 2018). The functional and integrated stroma and ECM depend on complicated interactions and networks between collagen fibrils, cellular interfaces, and non-fibrillar collagens macromolecules, including proteoglycans, glycoproteins, GAGs, and fibrillin (Isaacson et al., 2018). A comprehensive decellularized model should aptly mimic all components with the native corneas, as we have attempted to illustrate below.

In the above section, a detailed outline of the human native corneas has been provided, illustrating the complexity of each layer’s structure and function. Thus, to create an ideal keratoprosthesis, the scaffold should mimic the intrinsic organization and components of native corneas. Therefore, we hypothesized a model generated through 3D bioprinting and decellularization. In general, we propose to utilize the Simplify3D^®^ Desktop Software to generate multilayered scaffolds (Griffith et al., 2016), while the decellularized corneal scaffolds can be used as the major constituents of the bioink. The reasons for combining these two powerful approaches are based on the fact that advances in tissue engineering and regenerative medicine have been accomplished by bioprinting layered structures using bioinks composed of various cells, polymers, and molecules (Gu et al., 2020). Simplify3D^®^ Desktop Software is well-suited for this complex manufacturing process. Additionally, success with this process depends heavily on the bioink.

**FIGURE 3:**
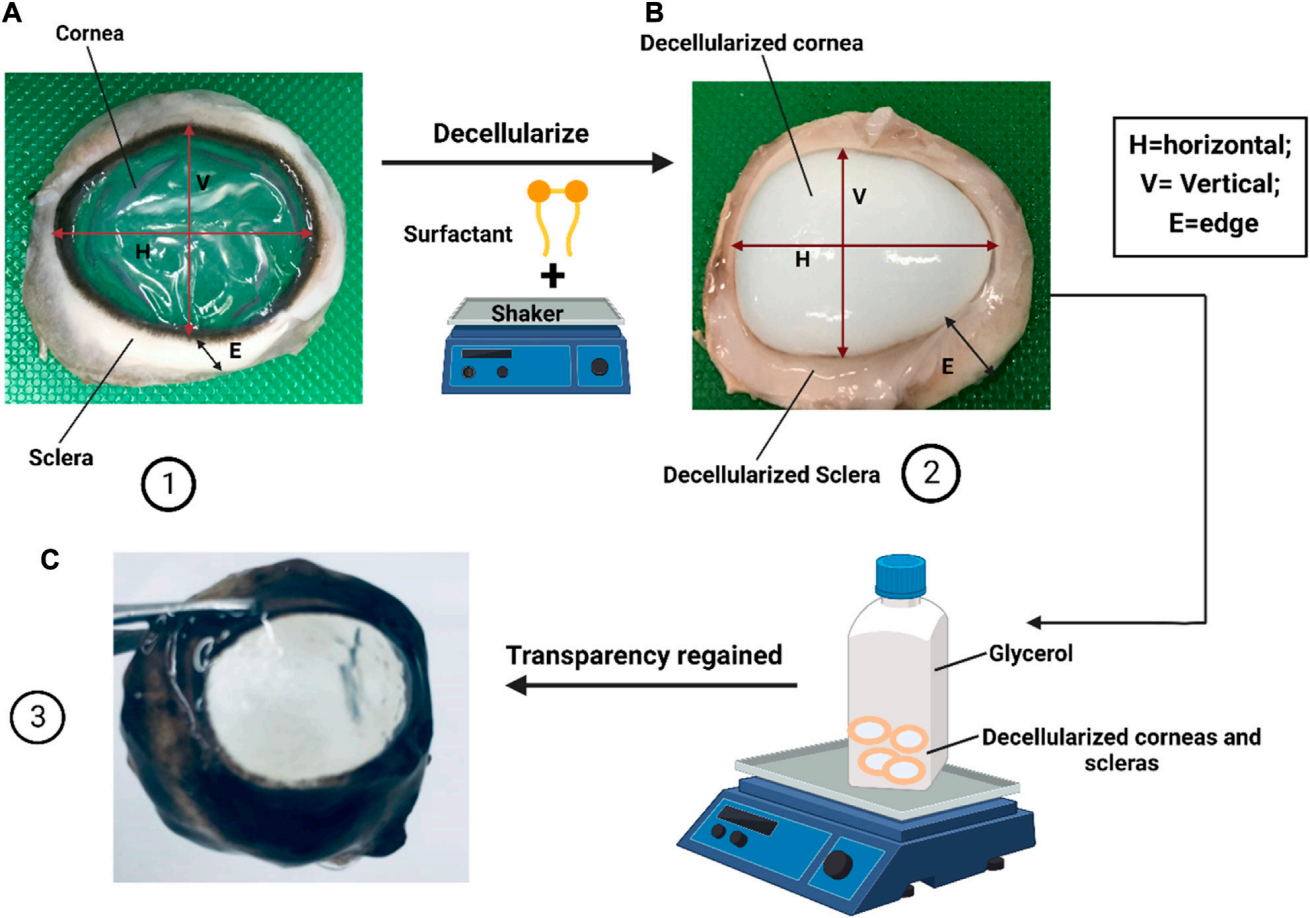
**(A)** Native ovine cornea; **(B)** Decellularized ovine cornea; **(C)** Decellularized cornea regained transparency.

**FIGURE 4:**
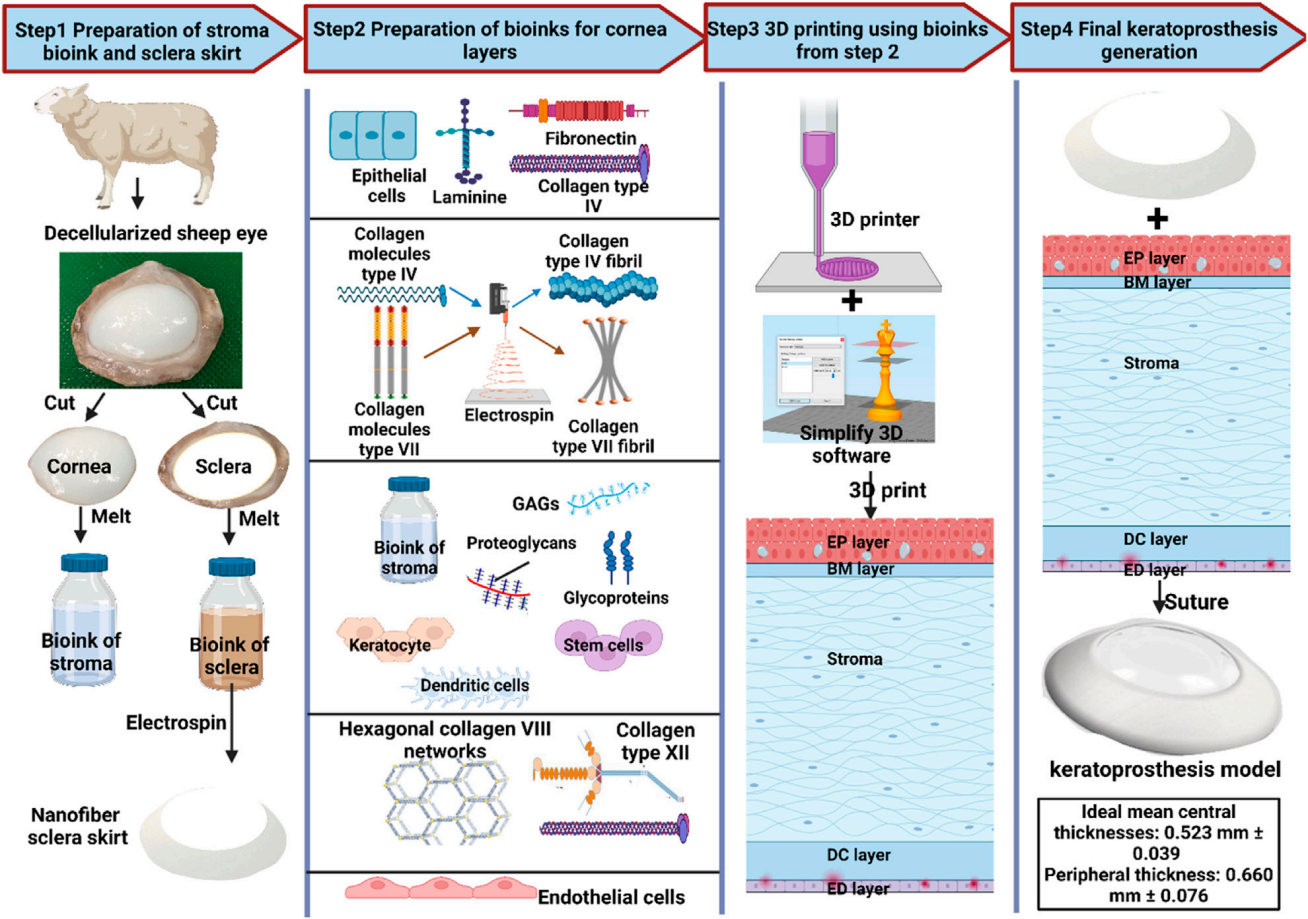
Idea model for multilayered keratoprosthesis. EP = epithelial; BM = Bowman’s layer; DC = Descemet’s membrane; ED = endothelial.

Ideally, decellularization will remove unwanted cellular materials without harming GAGs, proteoglycans, collagen, and other acellular parts (Du and Wu, 2011). Therefore, we assumed that our decellularized corneal stroma maintained these components whose crucial functions have been explained before. The workflow of the model has been demonstrated in Figure 4. The first step is to generate the decellularized cornea stroma and use these tissues to generate a viable bioink and decellularized sclera as a nanofiber skirt. The decellularized scaffolds are resourced from discarded native sheep corneas, which can be obtained in a limitless supply from local abbatoirs. Native sheep corneas have horizontal and vertical diameters of 2.50 cm and 1.79 cm, respectively, and a thickness of 0.63 mm post-decellularized via a combination of chemical (surfactants) and physical (agitation) processes (Nagata et al., 2010). The width of the cut sclera edge for native corneas was around 3.00 mm. The exact dimensions of DCs can be roughly 2.20 cm, 1.55 cm, 5.00 mm, and 3.98 mm, as outlined in Figure 3. It is noticed that decellularized ovine corneas are opaque, but their transparency will be significantly recovered by treatment with glycerol.

Specifically, we propose to use the DCs as a stromal layer bioink. Each scaffold in the large batch of DCs can be divided into the cornea and sclera. Both will be melted to form a gel or liquid. This procedure can be achieved by generating hydrogels from decellularized tissues via enzymatic and acidic digestions (Kort-Mascort et al., 2023). It has been shown that collagen type I can change into liquid crystal phases when concentrated in an acid solution, which may be a limiting factor (Giraud Guille et al., 2005). Then the liquified sclera can be electrospun into a nanofiber skirt for suturing and anchoring the graft. Afterward, the second step is to prepare bioinks for the 5 scaffold layers. The bioinks of the epithelial layer include collagen type IV, laminin, and fibronectin, while that of Bowman’s layer are made of collagen fibrils (type IV and VII) around 18–22 nm, which can be obtained via electrospinning.

The Descemet membrane’s ink should be composed of hexagonal collagen VIII networks and associated collagens IV and XII (Fitch et al., 1990; Hemmavanh et al., 2013), while the endothelial layer could be primarily composed of endothelial cells. Moreover, the stroma bioinks can be rich in collagen type I (Olivero and Furcht, 1993), GAGs, glycoproteins, proteoglycans, keratocytes, stem cells, and dendritic cells. The cells in bioinks can be cell-sheeted or printed into sheets. Following the complete preparation of bioinks, All the other components will be added into the extruder and printed layer by layer. The final printed product can be designed to possess mean central and peripheral thicknesses of 0.523 mm ± 0.039 and 0.660 mm ± 0.076, respectively, which correspond to the dimensions of the human cornea (Martola and Baum, 1968). After that, the bioink of the sclera, which is made of electrospun nanofibers and decellularized ovine materials, can be printed separately onto a nanofiber skirt that will be sutured using biocompatible threads to the cornea. Figure 4 provides an outline of the proposed model.

## Data Availability

The original contributions presented in the study are included in the article/supplementary material, further inquiries can be directed to the corresponding author.
